# Modular networks and genomic variation during progression from stable angina pectoris through ischemic cardiomyopathy to chronic heart failure

**DOI:** 10.1186/s10020-022-00569-3

**Published:** 2022-11-26

**Authors:** Lin Chen, Ya-Nan Yu, Jun Liu, Yin-ying Chen, Bo Wang, Yi-Fei Qi, Shuang Guan, Xi Liu, Bing Li, Ying-Ying Zhang, Yuanhui Hu, Zhong Wang

**Affiliations:** 1 Institute of Basic Research in Clinical Medicine, China Academy of Chinese Medical Sciences, No. 16 Nanxiaojie, Dongzhimennei, Beijing, 100700 China; 2 The Second Affiliated Hospital of Zhejiang Chinese Medical University (Zhejiang Xinhua Hospital), Zhejiang Chinese Medical University, Hangzhou, 310053 China; 3 Guang’anmen Hospital, China Academy of Chinese Medical Sciences, No. 5 Beixian Ge, Xicheng District, Beijing, 100053 China; 4 Xi Yuan Hospital, China Academy of Chinese Medical Sciences, No. 1, Xiyuan Playground, Haidian District, Beijing, 100091 China; 5 Institute of Chinese Materia Medica, China Academy of Chinese Medical Sciences, Dongzhimen, Beijing, 100700 China; 6 Geriatric Department, Dongzhimen Hospital Affiliated to Beijing University of Chinese Medicine, Dongzhimen, Beijing, 100007 China; 7 Department of Cardiovascular Diseases, Guang’anmen Hospital, China Academy of Chinese Medical Sciences, Beijing, China

**Keywords:** Modular networks, Genomic variation, Module reconstruction pairs

## Abstract

**Background:**

Analyzing disease–disease relationships plays an important role for understanding etiology, disease classification, and drug repositioning. However, as cardiovascular diseases with causative links, the molecular relationship among stable angina pectoris (SAP), ischemic cardiomyopathy (ICM) and chronic heart failure (CHF) is not clear.

**Methods:**

In this study, by integrating the multi-database data, we constructed paired disease progression modules (PDPMs) to identified relationship among SAP, ICM and CHF based on module reconstruction pairs (MRPs) of *K*-value calculation (a Euclidean distance optimization by integrating module topology parameters and their weights) methods. Finally, enrichment analysis, literature validation and structural variation (SV) were performed to verify the relationship between the three diseases in PDPMs.

**Results:**

Total 16 PDPMs were found with *K* > 0.3777 among SAP, ICM and CHF, in which 6 pairs in SAP–ICM, 5 pairs for both ICM–CHF and SAP–CHF. SAP–ICM was the most closely related by having the smallest average *K*-value (*K* = 0.3899) while the maximum is SAP–CHF (*K* = 0.4006). According to the function of the validation gene, inflammatory response were through each stage of SAP–ICM–CHF, while SAP–ICM was uniquely involved in fibrosis, and genes were related in affecting the upstream of PI3K–Akt signaling pathway. 4 of the 11 genes (FLT1, KDR, ANGPT2 and PGF) in SAP–ICM–CHF related to angiogenesis in HIF-1 signaling pathway. Furthermore, we identified 62.96% SVs were protein deletion in SAP–ICM–CHF, and 53.85% SVs were defined as protein replication in SAP–ICM, while ICM–CHF genes were mainly affected by protein deletion.

**Conclusion:**

The PDPMs analysis approach combined with genomic structural variation provides a new avenue for determining target associations contributing to disease progression and reveals that inflammation and angiogenesis may be important links among SAP, ICM and CHF progression.

**Supplementary Information:**

The online version contains supplementary material available at 10.1186/s10020-022-00569-3.

## Background

Disease–disease relationships play crucial roles in pathobiological manifestations of diseases and precision treatment to managing those conditions. Therefore, exploring associations of diseases enhances knowledge of disease relationships, which could further improve approaches to disease diagnosis, prognosis, and treatment (Iida et al. 2020; Suratanee and Plaimas 2015). Like other complex diseases, stable angina pectoris (SAP), ischemic cardiomyopathy (ICM) and chronic heart failure (CHF) are caused by interactions of environmental factors and genetic (Dang et al. 2020). SAP is a chronic medical condition which is generally regarded as one of the first manifestations or warning signs of underlying coronary artery disease (CAD), with an annual mortality rate ranging between 1.2 and 2.4% (Gillen and Goyal 2021; Montalescot et al. 2013). When ICM describes ineffective blood pumping by the heart as a result of ischemic damage to the myocardium, which is most often caused by CAD (Bhandari et al. 2021; Sekulic et al. 2019). In addition, Heart failure (HF) as the terminal state of various heart diseases with a prevalence of around 26 million worldwide (Wolfson et al. 2018), and ICM is regarded as the leading cause, accounting for approximately for more than 60% of systolic HF cases in industrialized countries (Alimadadi et al. 2020). Moreover, some common pathological processes have been detected in SAP, ICM, and CHF: such as inflammation and oxidative stress (Daiber et al. 2021), microvascular dysfunction, cardiac ischemia (Tousoulis et al. 2013), extracellular matrix destruction with the participation of matrix metalloproteinases and other mechanisms are being discussed (Bansal et al. 2019; Chumakova et al. 2021). Therefore, there are disease progression process and causative links among the SAP, ICM and CHF. However, no studies have examined the relationships between these three diseases.

A network-based approach is useful for analyzing disease–disease relationships and many methods are derived (Iida et al. 2020), such as MiRNA-disease Association Prediction method (Sumathipala and Weiss 2020), dynamic network biomarker method (Yang et al. 2018), and meta-path-based Disease Network capturing algorithm method et al. (Jin et al. 2019). Modularity, one of the most significant global characteristics of biological networks, has been the subject of intense investigation in systems biology for more than two decades (Grunberg and Del Vecchio 2020; He et al. 2019). This principle is important because it helps to account for the robustness and reliability of biological systems (Kashtan and Alon 2005). Recently it has been studied that similar genetic diseases could appear as modules in a human disease network (Ni et al. 2020). The modularity significantly correlated with disease classification, that is, disease phenotypes within a single module tended to fall in the same disease class (Jiang et al. 2008). Evidence from many sources suggests that diseases with overlapping clinical phenotypes are caused by mutations of functionally related genes (Brunner and van Driel 2004). Functionally related genes generally indicate genes which belong to the same functional modules, such as co-expression modules, protein complexes or cellular pathways. The exploration of modular structure has been a key factor in understanding the complexity of disease networks (Chen et al. 2016). There is growing evidence that modular units of development were highly preserved and recombined during evolution (Lacquaniti et al. 2013). With the rapid progress in probing into the detailed structural model of modular networks, flexible modular organization manifests a key adaptive balancing ability of allosterically regulating or reconstructing intermodular and intramodular states to uncover the novel biological alterations beyond engineering properties (Bowsher 2011; Del Mondo et al. 2009; Yu et al. 2016). In our previous study, we proposed the concept of restructured modules (RMs) was defined as those with larger architectural variation to quantifying the polyphyletic modular flexibility (Patent No: ZL201610826031.1) (Yu et al. 2016). The RMs may provide valuable structural change information about disease network. Therefore, disease progress relationships among the three diseases may be identified using this methods. In addition, genomic variability is a window on the origins of complex disease, cardiovascular disease in particular (Erdmann et al, 2018; Lin 2021). To understand the mechanisms of diseases, find pathogenic targets, and carry out personalized precision medicine, it is critical to detect such variations (Bennett et al. 2019; De et al. 2019). Thus we included modular networks and genomic structural variation (SV) information in the models to determine target associations contributing to the disease progress.

In our research, by integrating the multi-database data, we constructed paired disease progression modules (PDPMs) to identified relationship among SAP, ICM and CHF based on RMs methods. The relevance of the identified PDPMs with the diseases was validated by pathway enrichment analysis, functional analysis and literature. The understanding of mechanisms linking SAP, ICM and CHF progression is crucial for identifying specific actionable therapeutic targets.

## Methods

### Constructing disease‑associated networks

List of disease-related genes were obtained from National Center for Biotechnology Information (NCBI) database (https://www.ncbi.nlm.nih.gov/) and Genecards database (https://www.genecards.org/). We used disease-associated genes to construct three disease-associated networks using the STRING 11.5 database (https://string-db.org/). Cytoscape software v3.8.2 (https://cytoscape.org/) was utilized to visualize the networks and analyze the network parameters.

### Identification of functional modules in different disease‑associated networks

Network module division was performed using “MCODE”, “Community Clustering (GLay)”, and “MCL” (Chen et al. 2021). For MCODE, the parameters (Node Score Cutoff = 0.2, Node Score Threshold = 0.2, Connectivity Threshold = 2, Degree Cutoff = 2, Core Threshold K = 2, Max. Depth = 100) were used as the criteria for network module screening (Liu et al. 2019). We calculated the entropy of the network to select approach with the minimum entropy to divide the three disease-connected networks into modules (Chen et al. 2021).

### Identifying modules reconstructional pairs (MRPs)

The MRPs was found based on the overlapping nodes between each two disease modules. For example, for SAP- vs ICM-associated networks, (1) Overlapping nodes were detected between SAP- and ICM-disease modules. (2) We call a module pair with at least one node overlapping as a MRP.

### Calculating the *K*-value of the MRPs

The degree of reconstruction of MRPs between diseases was assessed with the *K*-value, which is based on Euclidean distance optimization (Liu et al. 2021).

Step 1: Firstly, non-dimensionalize the raw data. Due to the large dimensional differences between different indicators, we need to standardize the raw data first. In this study, the values of average neighbor nodes, characteristic path lengths, nodes and edges need to be normalized to between 0–1. Following the extreme value method in Gregory and Jackson (Gregory and Jackson 1992), we use it as the method of non-dimensionalize, which is shown in Eqs. (1) and (2) below (Wang et al. 2021b).

1 $$\mathrm{Cost\, index}: Di=\frac{f{i}_{max}-fi}{f{i}_{max}-f{i}_{min}}$$

2 $$\mathrm{Benefit \,index}:Di=\frac{fi-f{i}_{min}}{f{i}_{max}-f{i}_{min}}$$

Step 2: Considering the different meaning and multiplicity of each variable and eliminating the heterogeneity caused by multidimensional, we calculate the relative distance ${d}_{i}$ of each variable.

3 $${d}_{i}=\frac{{\left({a}_{i}-{b}_{i}\right)}^{2}}{{\left({a}_{1}-{b}_{1}\right)}^{2}+\dots +{\left({a}_{n}-{b}_{n}\right)}^{2}}$$

Step 3: The entropy weight method measures the amount of information provided by each index from a mathematical point of view and determines the weight of each index on this basis. As an objective weighting method, it can reduce the interference from human factors on the evaluation results, scientifically calculate the entropy weight of each index, and produce more scientific evaluation results (Wang et al. 2021a). The SPSSAU project (Version 21.0), an online application software retrieved from https://www.spssau.com, was used to calculate weight vector by entropy (Lin et al. 2020).

Step 4: Based on the module topology parameters and the determination of their weights, we calculated *K*-value follow Eqs. (4)

4 $$K=\sqrt{{w}_{1}{d}_{1}+\cdots{w}_{n}{d}_{n}}$$

### Identifying paired disease progression modules (PDPMs)

We calculated the *K*-value statistical distribution ($${k}_{i})$$ between modules in the range of 0–100% follow Eqs. (5). Previous studies have shown that the golden section method with fast convergence is a classical algorithm in optimization calculation, which is famous for its simplicity and remarkable effect (Santos et al. 2020; Julong and Fucai 2005). Therefore, we took the golden section method (61.8%) as the dividing point to divide the $${k}_{i}$$. PDPMs were defined more than 61.8% of the $${k}_{i}$$ of MRPs.

5 $${k}_{i}=\frac{{k}_{i}-{k}_{min}}{{k}_{max}-{k}_{min}}\times \%$$

### Functional enrichment analysis of PDPMs

The enrichment analysis of KEGG pathways and biological processes in the modules and disease-related genes was performed using metaspace (https://metascape.org/). (on Aug. 1, 2021) (Min Overlap: 3; P Value Cutoff: 0.01; Min Enrichment 1.5) (Zhang et al. 2020).

### Validation of overlapping genes in the PDPMs by text mining in the literature

We used PubMed database and CTD database to verify the overlapping genes in the PDPMs by searching the literature with the terms “stable angina pectoris”, “ischemic cardiomyopathy”, “chronic heart failure” and “gene ID”.

### Database of genomic variants

For information on the genomic structural variation observed in the population, we used the Database of Genomic Variants (DGV) (http://dgv.tcag.ca/dgv/app/home). DGV provides high‐quality structural variations, defined as a region of DNA elements approximately 1 kb and larger and can include inversions and balanced translocations or genomic imbalances (insertions and deletions), commonly referred to as copy number variants. The content of DGV represents SV identified in healthy control samples from large published cohorts and integrated by the DGV team (Dafniet et al. 2020). We worked with the latest release available from the GRCh37 (hg19) assembly of supporting variants section and gnomAD_Structural_Variants study (on January 21, 2022). We extracted SVs with variant subtypes, including “complex”, “inversion”, “loss” and “gain”. SVs with unknown information were removed.

## Results

### Disease-related targets

**Fig. 1:**
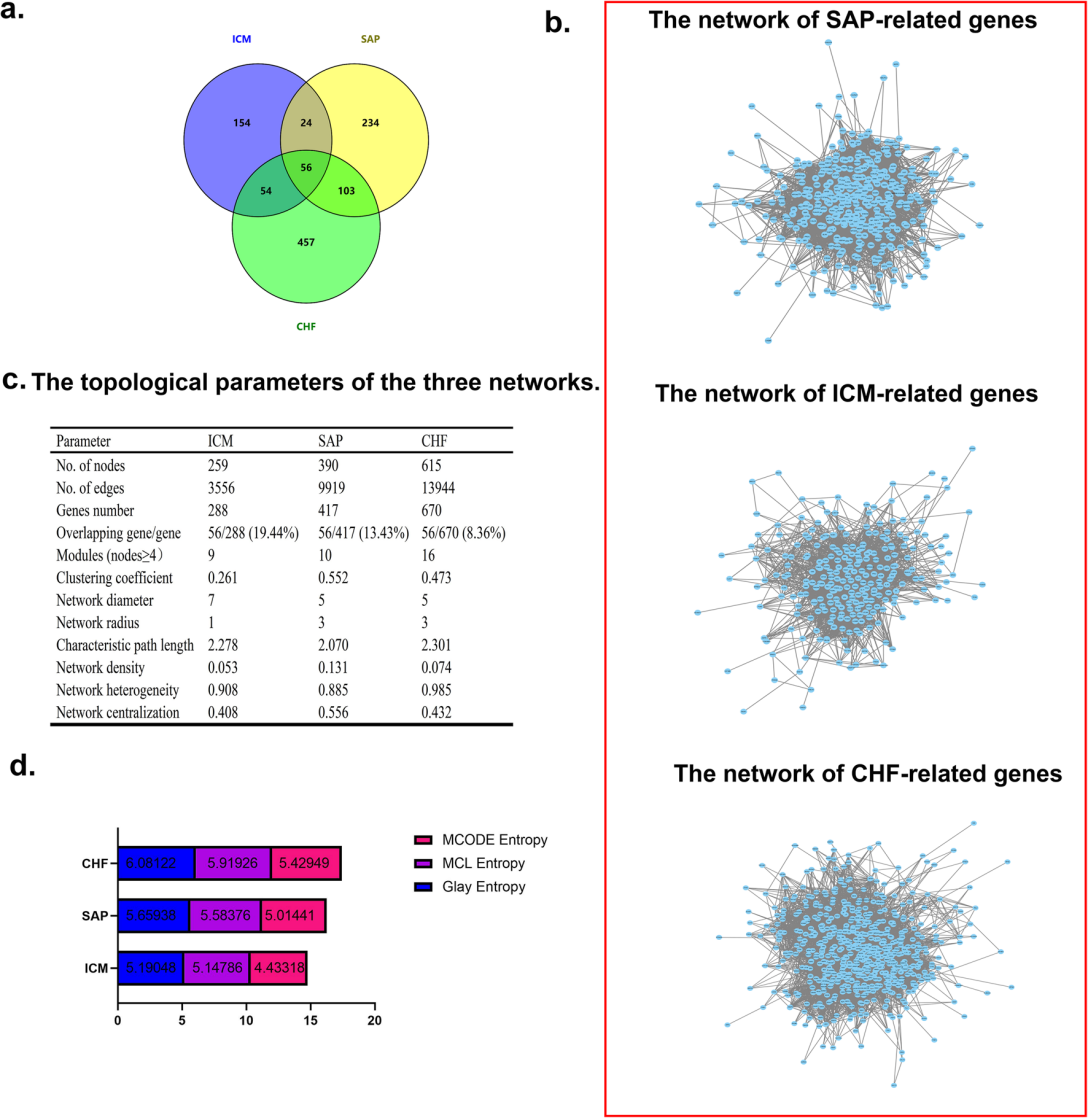
Comparison of disease-related genes and network analysis in SAP, ICM, and CHF. **a** Comparison of disease-related genes in SAP, ICM, and CHF. **b** The network of SAP-, ICM- and CHF-related genes. **c** Topological parameters of the three networks. **d** The entropy calculation of the network in SAP, ICM, and CHF

From the NCBI database and the Genecard database, a total of 288 ICM disease-related proteins, 417 SAP disease-related proteins and 670 CHF disease-related proteins were obtained (Additional file 1: Table S1). 56 overlapping genes were detected among three diseases, which accounted for 19.44% (56/288) of the identified ICM-associated genes, 13.43% (56/417) of SAP-associated genes, and 8.36% (56/670) of CHF-associated genes. In addition, 24 overlapping genes were detected between ICM‐ and SAP‐related genes, 54 between ICM- and CHF-related genes, and 103 between SAP- and CHF-related genes, respectively (Fig. 1a, Additional file 2: Table S2).

### Constructing disease-associated networks

ICM-, SAP-, and CHF-associated PPI networks were constructed, involving 259, 390, and 615 nodes, respectively (Fig. 1b). The multiple topological parameters of the three disease networks are listed in (Fig. 1c). CHF-associated networks contained the maximum number of nodes (genes) and edges (interactions). However, the SAP-associated network had the biggest network density (0.131), network centralization (0.556) and clustering coefficient (0.552). Therefore, an analysis of the entire network might not be sufficient to reveal the pathophysiological changes among the three diseases.

### Identification of functional modules

**Fig. 2:**
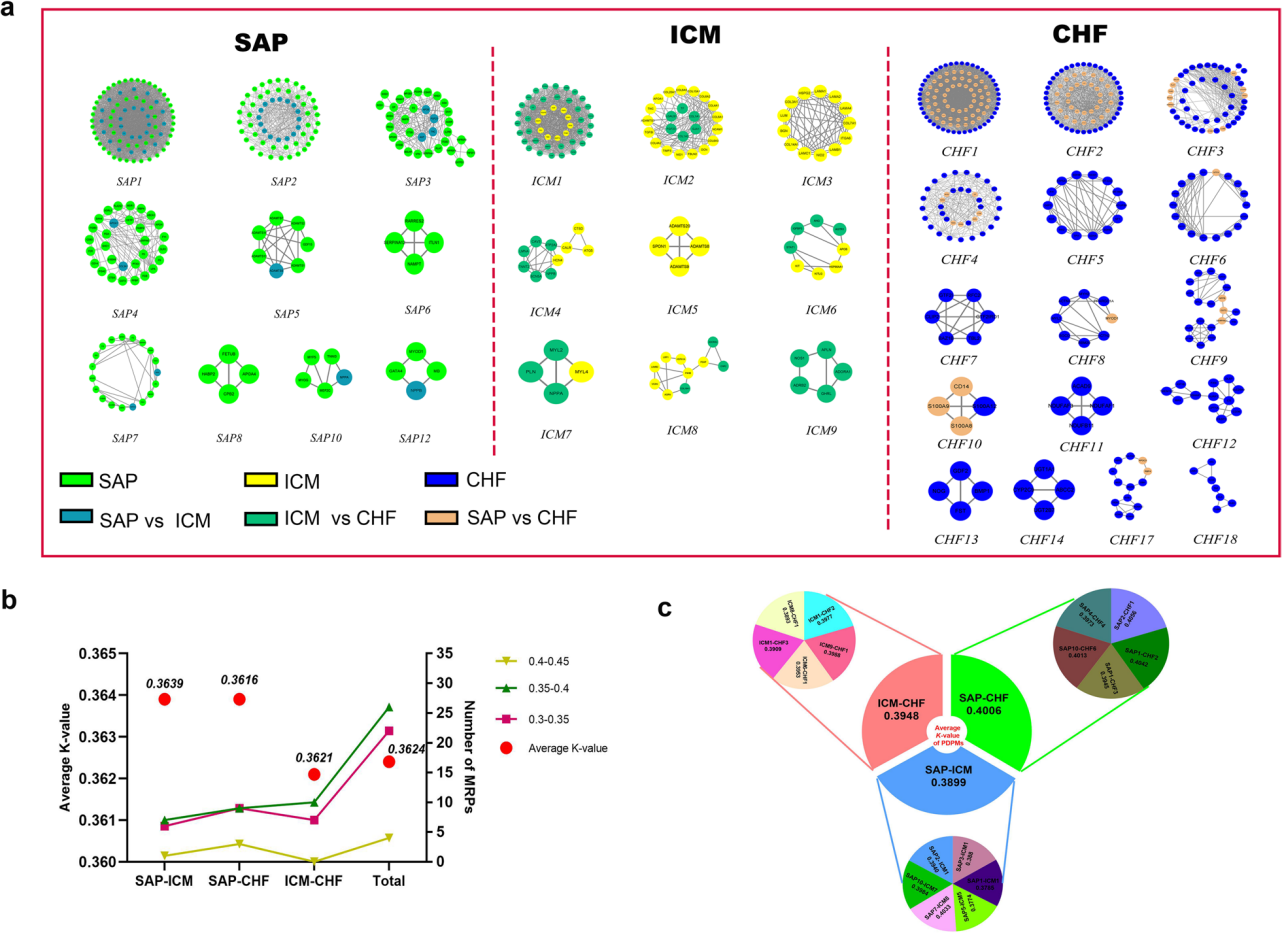
Identification of module and module reconstructional pairs. **a** Identification of module and module reconstructional pairs. **b** The distribution of the *K*-value. **c** The distribution of the *K*-values of the PDPMs

We selected MCODE with the minimum entropy among the three disease-networks to divide the three disease-connected networks into modules (Fig. 1d). 9, 10, and 16 modules (nodes ≥ 4) were identified from ICM-, SAP-, and CHF-associated networks, respectively (Fig. 2a). Module details were provided in Additional file 2: Table S3. Topological attributes of disease-associated modules are provided in Additional file 2: Table S4.

### Identification of MRPs

Figure 2A shows the results of MRPs. Compared with ICM-associated modules, SAP-associated networks had eight reconstructional modules. ICM-associated networks had seven reconstructional modules and the *ICM9* module was new module compared with CHF-associated modules. Compared with SAP-associated networks, nine reconstruction modules are in CHF-associated modules.

The MRPs among ICM-, SAP- and CHF-associated networks were shown in Additional file 2: Table S5 in detail. There were 14 MRPs between ICM- and SAP-related modules, 21 between SAP- and CHF-related modules and 17 MRPs between ICM- and CHF-related modules. We found that the splitting and merging between modules occurred in the process of disease progression. For example, the *ICM9* module has five nodes, which are scattered among the *CHF1*, *CHF2* and *CHF4* modules. We considered that this change was the splitting of modules in the disease progression. Three nodes of the *CHF10* module are from the *SAP4* and *SAP7* modules, which was considered to be the merging of some nodes of several modules of SAP.

### Quantitative comparative analysis of module reconstruction of *K*-value model

The *K*-values of MRPs among the three diseases are show in Fig. 2b. In general, the *K*-value was mainly distributed between 0.35–0.4, and the average *K-*value of each stage was approximately 0.36. Additional file 2: Table S6 shows the weight values calculated by entropy weight method. The details of the normalization of disease-related module parameters and *K*-values are shown in Additional file 2: Tables S7 and S8, respectively.

### Identification of PDPMs

The smaller the *K*-value is, the smaller the overall difference between the two modules, and the two modules are similar in structure. Combined with the statistical distribution and golden section method, it is considered that *K* > 0.3777 and was PDPMs (Additional file 2: Table S8). Finally, sixteen PDPMs were involved, specifically 6 pairs in SAP–ICM and 5 pairs each for ICM–CHF and SAP–CHF. SAP–ICM was more closely related by having the smallest average *K*-value (*K* = 0.3899), followed by ICM–CHF (*K* = 0.3948) and SAP–CHF (*K* = 0.4006) (Fig. 2c). Moreover, *PDPM*_SAP7–ICM8_, *PDPM*_ICM9–CHF1_ and *PDPM*_SAP2–CHF1_ were the PDPMs with the largest *K*-value in the SAP–ICM, ICM–CHF and SAP–CHF stages, respectively.

**Fig. 3:**
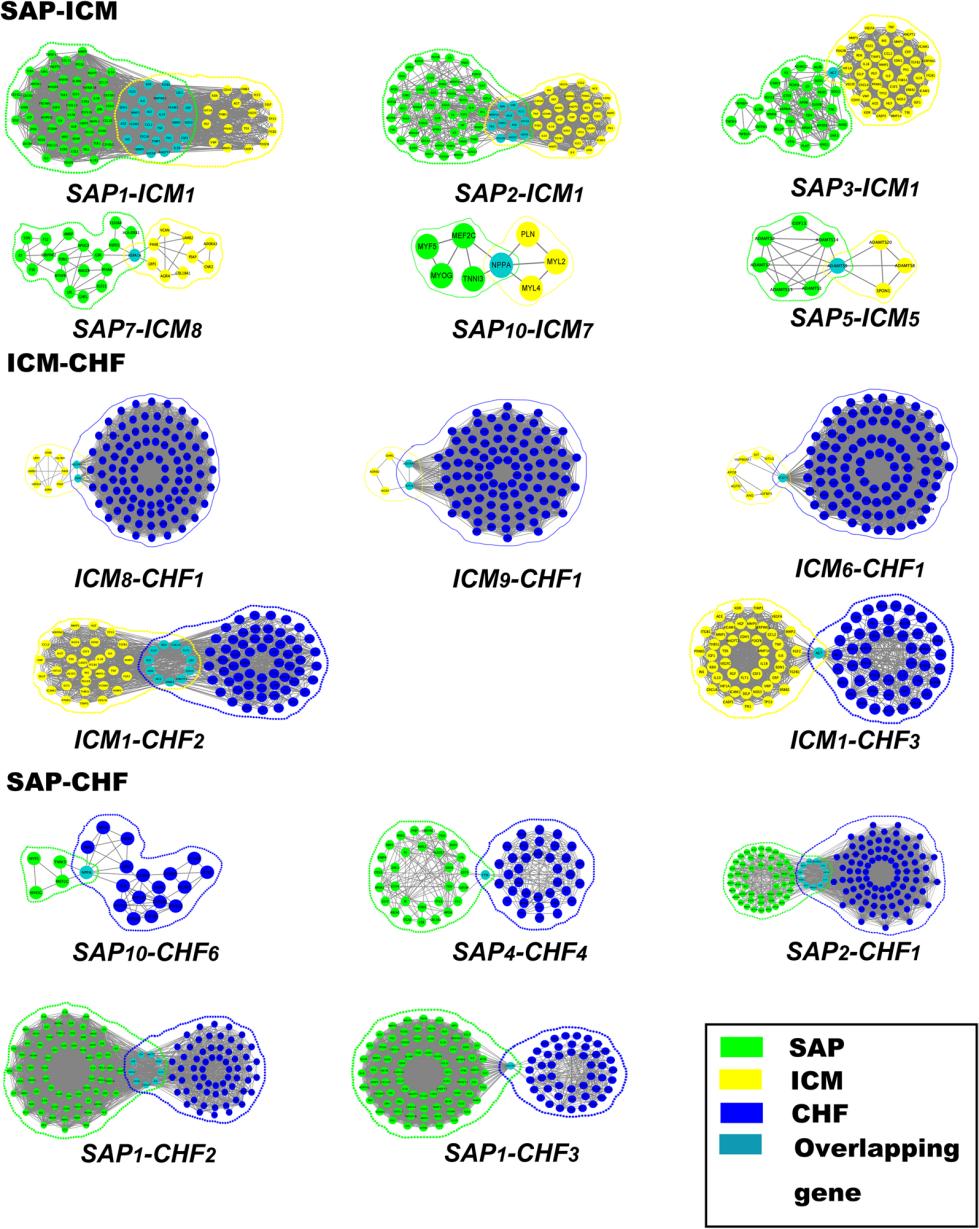
Identification of paired disease progression modules

In the progress of SAP–ICM–CHF, modules ICM1 and ICM8 are common modules, which involve five genes affecting the whole process (AGT, REN, CDH5, PGF, and FLT1), of which modules ICM5 and ICM7 are specific to the SAP–ICM, involving ADAMTS9 and NPPA, modules ICM6 and ICM9 are specific to ICM–CHF, involving STAT1, ADORAL and APLN. At the same time, six genes were observed in three stages: ACE, CXCL8, IL10, CRP, KDR, and ANGPT2 (Fig. 3).

### GO functional enrichment analysis in SAP, ICM, and CHF

**Fig. 4:**
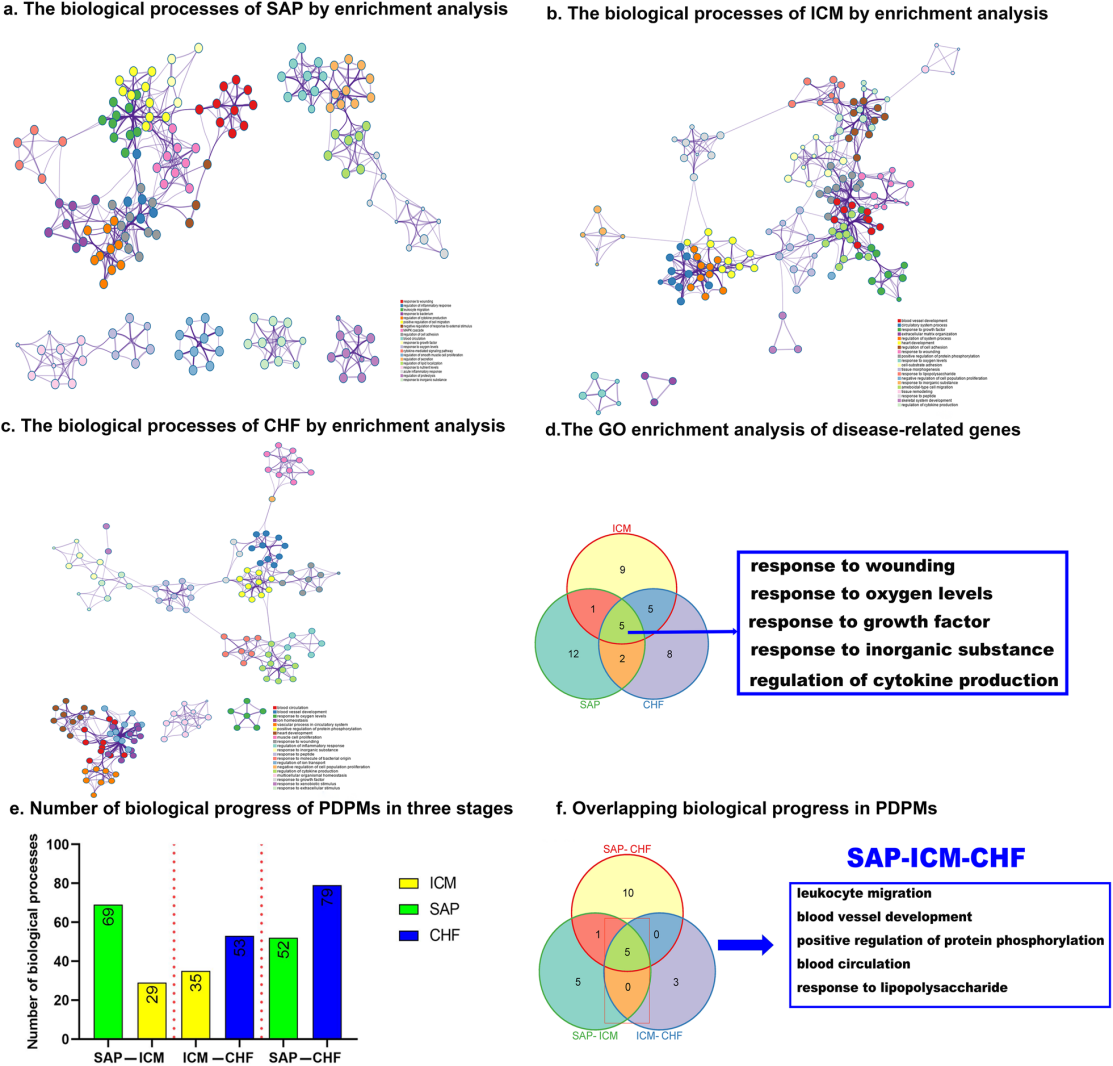
The biological process enrichment analyses of disease-related genes and PDPMs. **a** Top 20 biological process enrichment analyses of SAP-related genes. **b** Top 20 biological process enrichment analyses of ICM-related genes. **c** Top 20 biological process enrichment analyses of CHF-related genes. **d** Overlapping biological processes of three disease-related genes. **e** Number of biological processes of PDPMs in the three stages. **f** Overlapping biological progress in PDPMs

Based on SAP-, ICM-, and CHF-related genes, the top 20 GO biological process clusters with their representative enriched terms are shown in Fig. 4. In SAP, response to wounding, regulation of inflammatory response and leukocyte migration were the top three functional biological processes (Fig. 4a). Blood vessel development, circulatory system process and response to growth factor accounted for most of the functional annotations in ICM (Fig. 4b). In CHF, blood circulation, blood vessel development and response to oxygen levels were noted to be the major functional annotations (Fig. 4c). Response to wounding, response to oxygen levels, response to growth factor, response to inorganic substance and regulation of cytokine production were the overlapping GO biological processes shared by all three diseases (Fig. 4d).

In the 16 PDPMs, SAP-related modules enriched 69 and 52 biological processes in two different stages, 29 and 36 in ICM-related modules, 53 and 79 in CHF-related modules (Fig. 4e).The number of overlapping biological processes between any two pathological stages (SAP–ICM, ICM–CHF and SAP–CHF) was 11, 8, and 16, respectively. A total of 5 overlapping biological processes were identified among the three pathological stages and SAP–ICM, while 3 were identified in ICM–CHF (Fig. 4f and Additional file 2: Table S9).

### KEGG pathway analysis in SAP, ICM, and CHF

**Fig. 5:**
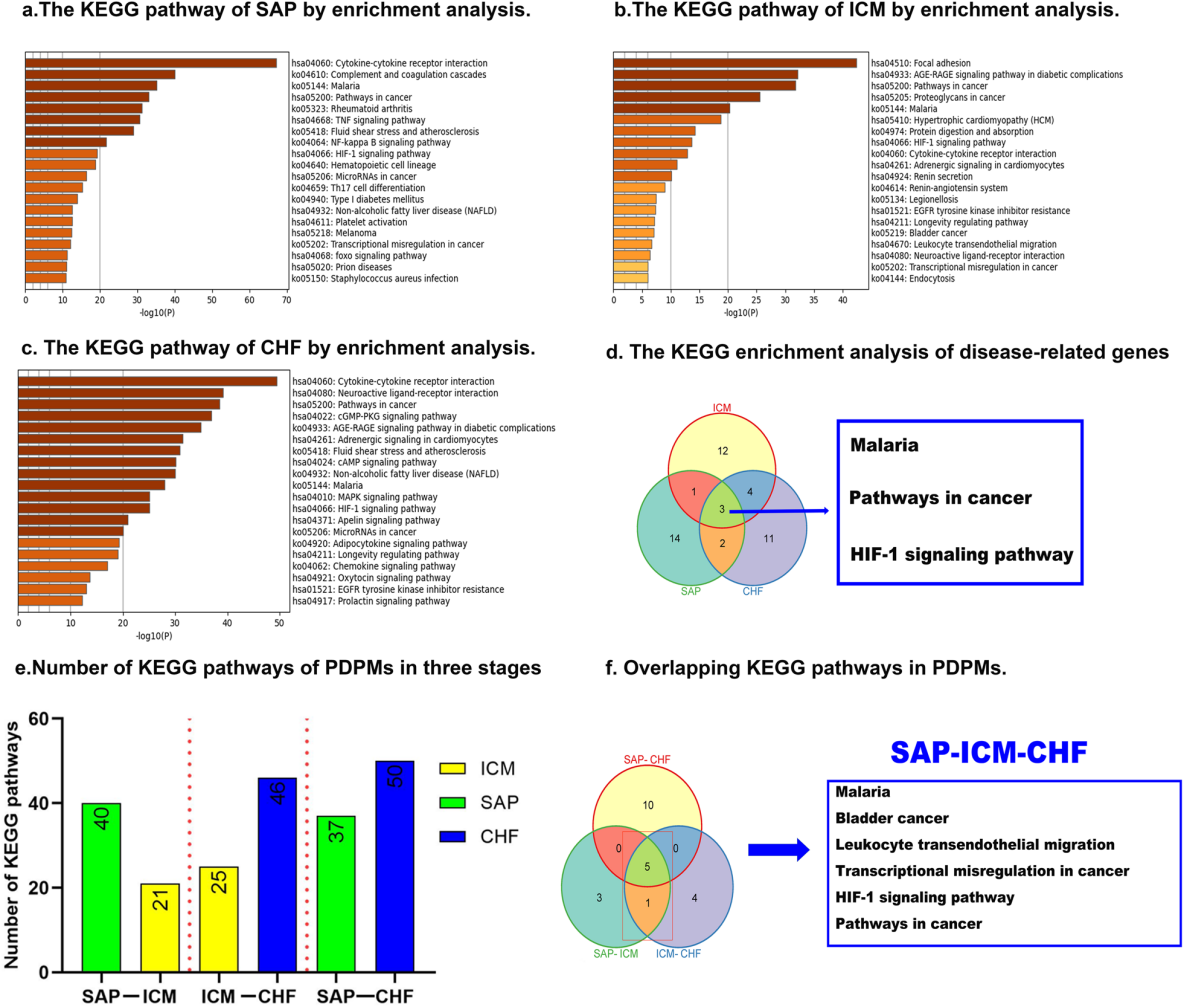
The KEGG pathways enrichment analyses of disease-related genes and PDPMs. **a** Top 20 KEGG pathways enrichment analysis of SAP-related genes. **b** Top 20 KEGG pathways enrichment analysis of ICM-related genes. **c** Top 20 KEGG pathways enrichment analysis of CHF-related genes. **d** Overlapping KEGG pathways of three disease-related genes. **e** Number of KEGG pathways of modules in three stages. **f** Overlapping KEGG pathways in PDPMs

Based on SAP-, ICM-, and CHF-related genes, the top 20 KEGG pathway clusters with their representative enriched terms are shown in Fig. 5. In SAP, cytokine–cytokine receptor interaction, complement and coagulation cascades and malaria were the top three pathways (Fig. 5a). Focal adhesion, the AGE-RAGE signaling pathway in diabetic complications and pathways in cancer accounted for most of the KEGG pathways in ICM (Fig. 5b). In CHF, cytokine–cytokine receptor interaction, Neuroactive ligand–receptor interaction and Pathways in cancer noted to be the major pathways (Fig. 5c). From previous studies, there were 6 pathways in the top 3 KEGG pathways for each disease reported the correlations as shown in the Additional file 2: Table S10. Malaria, pathways in cancer and the HIF-1 signaling pathway were the overlapping KEGG pathways shared by all three diseases (Fig. 5d).

In the 16 PDPMs, SAP-related modules enriched 40 and 37 pathways in two different stages, 21 and 25 in ICM-related modules, 46 and 50 in CHF-related modules (Fig. 5e).The number of overlapping KEGG pathways between any two pathological stages (SAP–ICM, ICM–CHF and SAP–CHF) was 9, 10, and 15, respectively. A total of 6 overlapping pathways were identified among the three pathological stages, including 3 disease-related overlapping pathways, of which the HIF-1 signaling pathway contained the most overlapping genes for PDPMs (Fig. 5f and Additional file 2: Table S9).There were 3 and 4 unique KEGG pathways in SAP–ICM and ICM–CHF, respectively (Fig. 5f). In the 6 overlapping KEGG pathways in SAP–ICM–CHF in PDPMs, half of them have been reported the certain biological connections with the 3 diseases as shown in Additional file 2: Table S11.

### Validation of overlapping genes in the PDPMs based on a literature search

**Fig. 6:**
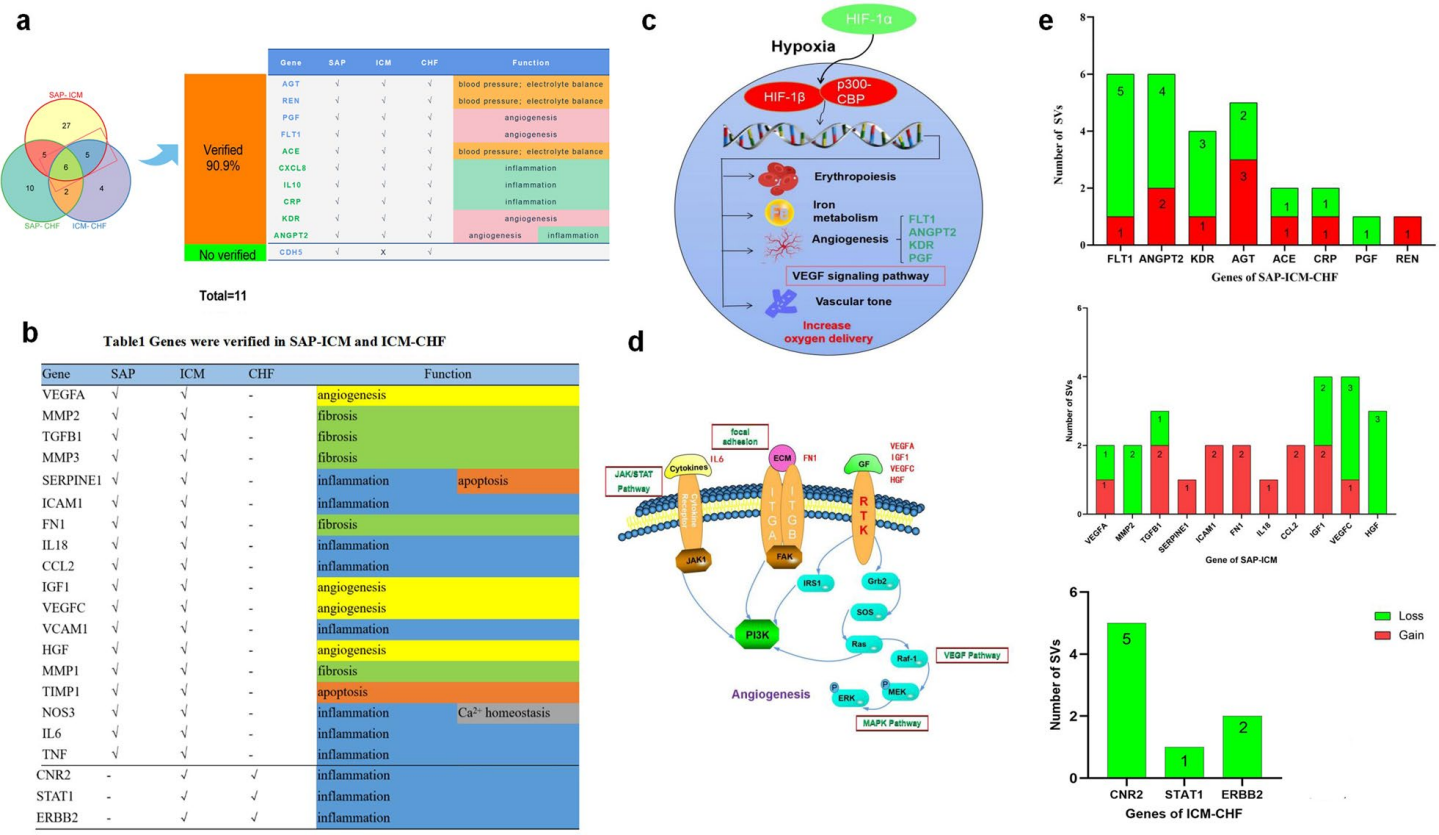
Literature verification and structural variations of overlapping genes in PDPMs. **a** Literature validation of overlapping genes in PDPMs. **b** Literature validation of overlapping genes of SAP–ICM and ICM–CHF in PDPMs. **c** HIF-1 signaling pathway and targets involved. **d** PI3K–AKT signaling pathway and targets involved. **e** Number of structural variations of verified overlapping genes in PDPMs

In our study, after verifying the overlapping genes in the PubMed database and CTD database, we obtained 10 validation genes except CDH5 (Fig. 6a). It is mainly divided into four parts according to the function: three genes belong to blood pressure and electronic balance, and 4 genes belong to angiogenesis and inflammation. A total of 66.67% (18/27) of genes were validated in SAP–ICM. In addition to inflammation and angiogenesis, fibrosis was also among the top three functions, while 75% (3/4) of the verified genes in ICM–CHF were mainly associated with inflammation (Fig. 6b). The HIF-1 signaling pathway is closely related to hypoxia–ischemia in cardiovascular diseases, and we found four genes (FLT1, KDR, ANGPT2, and PGF) related to angiogenesis in the pathway (Fig. 6c) (Zhang et al. 2018). The SAP–ICM genes mainly affects the upstream of the PI3K–AKT signaling pathway (Fig. 6d). Two of the four genes in ICM–CHF were involved in the Neuroactive ligand-receptor interaction signaling pathway.

### Structural variations (SVs) on targets associated with PDPMs

Matching the 10 verified genes of SAP–ICM–CHF with the data from the DGV, we identified 8 targets having SVs, 37.04% (10/27) SVs were defined as “gain” (replication of the protein) and 62.96% (17/27) SVs defined as “loss” (deletion of the protein). Four genes (FLT1, KDR, ANGPT2, and PGF) related to angiogenesis had 76.47% (13/17) of SVs were defined as “loss” and 23.53% (4/17) of SVs were defined as “gain”. Among the verified genes, 11 and 3 genes had SVs in SAP–ICM and ICM–CHF, respectively, and SAP–ICM involved 53.85% (14/26) “gain” SVs, while ICM–CHF is fully involved “loss” (Fig. 6e).

## Discussion

In this paper, we applied the restructured modules method to explore the dynamic evolution of disease progression and found 16 potential PDPMs among SAP, ICM, and CHF. The process of three diseases was closely related to ischemia and hypoxia, involving angiogenesis, inflammation, electrolytes and blood pressure factors at the levels of genes, biological processes and pathways. In genomic structural variation, we also found that overlapping genes of PDPMs involve more number of protein loss than protein replication.

Community structure is a special perspective for understanding the structures and functions of complex networks and can also significantly affect the dynamical behaviors on networks (Hu et al. 2020). It is well known that *Modularity* is one of the most commonly used methods to detect the community structures (Newman and Girvan 2004). Community or module can be helpful for identifying the disease genes and understanding the disease progression (Goh and Choi 2012). Thus, the progress of the three diseases was explored by reconstructing modules, establishing the relationship among the modules (finally forming PDPMs), and quantifying module changes with *K*-values, in which SAP–ICM is closest and ICM–CHF is close behind. Clinically, both SAP and ICM belong to ischemic heart disease, and myocardial ischemia makes the heart experience the pathological process from anginal pain to hibernating myocardium to cell death (Moroni et al. 2021). Myocardial hibernation is one of the main pathogenesis of ischemic cardiomyopathy (Frangogiannis 2003). Ischemic heart disease promotes complex inflammatory and remodeling pathways which contribute to the development of chronic heart failure (Dundas et al. 2021). Therefore, the relationship between the three diseases found by *K*-value is consistent with the association of pathological processes.

Module ICM1 and ICM8 are common modules were detected in SAP- ICM–CHF according PDPMs, and involve 11 genes affecting the whole process. Functions of overlapping targets can be classified into angiogenesis, inflammation, electrolytes and blood pressure. Multiple scientific reports point out that inflammation and angiogenesis are two interdependent processes underlying pathogenesis of cardiovascular disorders (Zernecke and Weber 2005; Skoda et al. 2018). Inflammatory cells secrete cytokines that activate ECs and stimulate their proliferation and migration, which constitute two characteristic steps of angiogenesis (Herrmann et al. 2006). PGF, as a member of the VEGFs family that binds two VEGF receptors (KDR and FLT1) (Carmeliet 2005), activates FLT1 in ECs inducing the expression of specific target genes (Kim et al. 2012). Moreover, studies found that increased expression of PGF was associated with the production of inflammatory markers such as CRP (Pilarczyk et al. 2008). In the course of disease progression, myocardial hypertrophy, fibrosis, and remodeling are involved, and a key mediator of this process is the activation of neurohormones, including regulators such as the renin–angiotensin–aldosterone system (Kitsios and Zintzaras 2007). AGT is well known to be the unique Ang I precursor. The inactive decapeptide Ang I is located at the N-terminus of AGT and, after its release by REN, is converted into the active octapeptide ANGPT2 by ACE. Inflammation regulates the renin-angiotensin system and blood pressure, but AGT inhibits angiogenesis (Corvol et al. 2003; Satou et al. 2018). Therefore, there are synergies and interactions between the overlapping targets of the three diseases and the functions of the targets.

According to the KEGG pathway analysis, we found that 4 gene expression (FLT1, KDR, ANGPT2, PGF) of HIF-1α-induced angiogenesis under hypoxia in HIF-1 signaling pathway affected the whole stage of SAP–ICM–CHF. The body can maintain the homeostasis of oxygen by activating HIF-1 signaling pathway in the hypoxic state and heart also needs a sufficient supply of oxygen to maintain effective contraction (Wei et al. 2012). Study confirms that low oxygenation concentrations in tissues (hypoxia) often trigger angiogenesis (Ramjiawan et al. 2017), which can be initiated independently of VEGF-related pathways, as well as lead to expression of multiple growth factors such as VEGF and ANGPT, via the HIF pathway (Cao et al. 2021). Numerous reports documenting HIF-1α up-regulation in response to mediators that are abundant in inflammatory conditions (Jung et al. 2003). In hearts manifesting pathological hypertrophy, the capillary density decreased during the transition from cardiac hypertrophy to heart failure (Flanagan et al. 1991). When capillary patterns were studied in histological sections, a significant decrease in capillary density was observed in the hearts of patients with ischemic cardiomyopathy (Karch et al. 2005). Moreover, the lack of HIF-1 will lead to angiogenesis disorder and myocardial fibrosis, resulting in heart failure (Tao et al. 2020). Thus, the imbalance of capillary angiogenesis is related to the transition process of ischemic cardiomyopathy and heart failure, and stimulating angiogenesis may be helpful to prevent or reverse heart failure (Oka et al. 2014).

Genomic variability, as it happens, is also the fuel of evolvability. Structural variation is one chapter in an evolving story and such variants dynamic, fluid and unstable (Gualtieri 2021). Identifying structural variation is essential for genome interpretation (Ho et al. 2020). Therefore, we need to mine structural variation information accurately. We found more protein deletions in the genes involved in SAP–ICM–CHF and ICM–CHF, particularly in the four genes involved in angiogenesis, while SAP–ICM involved more protein replication. Matsuoka et al. (2015) found that FLT1 may be susceptibility loci for miocardial infarction in Japanese individuals. Deletion polymorphism in the gene for ACE is a potent risk factor for myocardial infarction (Cambien et al. 1992). Those large (> 50 bp) regions of structural variation might impact the binding of the various proteins (Nanni et al. 2020). For example, they might remove or revert the nucleotide sequence, preventing the appropriate protein from recognising its motif and finally binding to the chain (Chiliński et al. 2022); therefore, we speculate that they may influence the gene binding of modules in the disease network, affecting disease progression.

Notably, our study has some limitations. First, the limitation of this study is the lack of independent validation. In addition, although we have selected two comprehensive and authoritative gene databases, we cannot guarantee the comprehensiveness of gene coverage. These issues needs to be addressed in our further studies.

## Conclusions

The PDPMs analysis approach combined with genomic structural variation provides a new avenue for determining target associations contributing to disease progression and reveals that inflammation and angiogenesis may be important links among SAP, ICM and CHF progression.

## Supplementary Information

**Additional file 1: Table S1.** Targets for ICM, SAP and CHF.

**Additional file 2: Table S2.** Related genes in the disease connection. **Table S3.** MCODE results for ICM-associated networks, SAP-associated networks, and CHF-associated networks. **Table S4.** Topological attributes of disease-associated modules (nodes ≥ 4). **Table S5.** Reconfiguration module matching among ICM-, SAP- and CHF-associated networks. **Table S6.** Weight results calculated by the entropy method. **Table S7.** Normalization of disease-related module parameters (nodes ≥ 4). **Table S8.** K-value of reconstruction module pairs among ICM, SAP and CHF disease (nodes ≥ 4). **Table S9.** Overlapping pathways and biological processes at each stage based on PDPMs. **Table S10.** The validation of top 3 pathways in three diseases from the literature. **Table S11.** Overlapping KEGG pathways in SAP–ICM–CHF in PDPMs.

## Data Availability

Not applicable.

## Abbreviations

SAP: Stable angina pectoris
ICM: Ischemic cardiomyopathy
CHF: Chronic heart failure
PDPM: Paired disease progression module
MRP: Module reconstruction pair
SV: Structural variation
CAD: Coronary artery disease
RM: Restructured module
