# Supplementary material for: Modular networks and genomic variation during progression from stable angina pectoris through ischemic cardiomyopathy to chronic heart failure
Source: Mol Med. 2022 Nov 26;28:140. doi: 10.1186/s10020-022-00569-3 (PMC9701405; doi:10.1186/s10020-022-00569-3)
Supplement: Supplementary file 2 — Additional file 2: Table S2. Related genes in the disease connection. Table S3. MCODE results for ICM-associated networks, SAP-associated networks, and CHF-associated networks. Table S4. Topological attributes of disease-associated modules (nodes ≥ 4). Table S5. Reconfiguration module matching among ICM-, SAP- and CHF-associated networks. Table S6. Weight results calculated by the entropy method. Table S7. Normalization of disease-related module parameters (nodes ≥ 4). Table S8. K-value of reconstruction module pairs among ICM, SAP and CHF disease (nodes ≥ 4). Table S9. Overlapping pathways and biological processes at each stage based on PDPMs. Table S10. The validation of top 3 pathways in three diseases from the literature. Table S11. Overlapping KEGG pathways in SAP–ICM–CHF in PDPMs. [file 10020_2022_569_MOESM2_ESM.docx]

| **Disease** | **Numbers** | **Disease-related genes** |
| --- | --- | --- |
| CHF  ICM  SAP | 56 | MMP2, XDH, EDN1, IGF1, NPPB, CD36, GHRL, TNNI3, AGT, HSPA4, CRP, CXCL8, NPPA, FABP3, ADRB1, TNF, IL1A, SELP, TNNT2, VWF, IL18, S100B, AGTR1, PTX3, VEGFA, TGFB1, TIMP1, CCL2, MMP1, IL6, BSG, ANGPT2, PARP1, ACE, MIR126, KDR, INS, FLT1, MMP3, PPARA, IL2RA, IL10, PGF, ADORA1, LGALS3, ICAM1, THBS1, NOS3, HADHB, NPPC, SERPINE1, VCAM1, CCN1, REN, CDH5, FGF23 |
| ICM  SAP | 24 | TEK, HSPD1, ADAMTS9, APOB, FN1, CTSB, MYF5, VEGFC, PDGFB, CSF3, AGER, VTN, HSPA1A, ANXA2, COL3A1, TNC, MMP14, FGF2, HIF1A, ADAMTS3, HCN4, KITLG, APOA1, HGF |
| CHF  ICM | 54 | RAC1, GJA1, COL1A1, PLN, DPP4, LGALS1, COL18A1, TF, CS, FLNC, PPARG, ST2, ALOX15, TCF4, ECE1, PPARGC1A, CMA1, CAV3, SPRED1, HOXA5, ACE2, ANG, ADRB2, CRAT, ADCY10, STAT1, CASP3, ALOX15B, TTR, UTS2, MIR130A, ERBB2, G6PD, PDE5A, ADORA3, IL6ST, MYH7, VCL, HFE, MKI67, FRZB, DMD, CCR5, MIR508, IGFBP3, LMNA, TIMP4, KCNJ11, MYL2, ATP2A2, NRG1, COL1A2, SCN5A, HMGB1 |
| CHF  SAP | 103 | CXCR4, CYBA, GDF15, CYCS, NOS2, SOD2, ITLN1, NLRP3, LEP, SMAD3, FOXP3, APOC3, APOE, ALDH2, FAS, SOD3, MIR223, MYH6, TNFRSF11B, MIR10A, CD40, CST3, CALCA, SERPINA1, IL1RL1, SELE, CNR1, CTF1, THBD, MYOD1, IL17A, BMP6, MPO, ELN, CASP1, SPP1, F2, PTGS2, OLR1, CTSK, S100A8, MIR21, CD14, HMGCR, MIR423, FASLG, CD163, IL1RAPL2, HMOX1, PECAM1, MYD88, BGLAP, RBP4, PLAT, TNFSF11, EGFR, MB, SOD1, CXCR3, IL2, IFNG, RETN, CXCL12, TNFRSF1B, APLN, TERT, PROM1, KNG1, IL1RN, MIR22, ADAMTS13, IL33, VASP, HLA-DRB1, LPL, CAT, ADAM17, FCN3, AHSG, TIMP2, IL1B, SLC24A3, LCN2, ALB, HAMP, SERPINC1, F3, S100A12, KCNJ5, CX3CR1, LTA, JUN, IL2RB, BAX, CP, CD40LG, AHSP, CYP3A4, GATA4, S100A9, MMP9, HBA1, ADIPOQ |

**Table S2 The Related genes in the Disease-connect**

**Table S3 MCODE results for ICM-associated networks, SAP-associated networks, and CHF-associated networks.**

| **ICM-associated networks** | | | | |
| --- | --- | --- | --- | --- |
| **Cluster** | **Score** | **Nodes** | **Edges** | **Genes** |
| 1 | 36.773 | 45 | 809 | ANGPT2, HGF, VCAM1, THBS1, TGFB1, MMP2, VEGFC, TIMP1, ACE, IGF1, PPARG, IL10, CXCL8, HIF1A, MMP3, TP53, ERBB2, MMP1, FGF2, VEGFA, TEK, KDR, PGF, ITGB1, REN, INS, ICAM1, CASP3, AGT, SELP, NOS3, FLT1, FN1, VWF, EDN1, CRP, CSF3, IL6, IL18, MMP14, PDGFB, TNF, CDH5, CCL2, SERPINE1 |
| 2 | 10.182 | 23 | 112 | NCAM1, FBLN2, GJA1, COL6A5, COL6A3, COL4A2, COL6A1, COL15A1, COL4A1, TF, TNC, LGALS1, COL20A1, TGFBI, COL1A1, FGF23, NID1, DCN, APOA1, COL1A2, ADAMTS3, COL6A2, TIMP3 |
| 3 | 9.167 | 13 | 55 | COL14A1, LAMA4, BGN, LAMB1, NID2, HSPG2, ITGA6, LUM, COL7A1, COL3A1, LAMC1, LAMA2, LAMA1 |
| 4 | 4.222 | 10 | 19 | NPPB, ATP2A2, CAV3, ATG5, LMNA, CTSD, SCN5A, HCN4, CALR, TNNT2 |
| 5 | 4 | 4 | 6 | SPON1, ADAMTS8, ADAMTS9, ADAMTS20 |
| 6 | 3.429 | 8 | 12 | HSP90AA1, IGFBP3, STAT1, AGTR1, KIT, APOB, KITLG, ANG |
| 7 | 3.333 | 4 | 5 | MYL2, MYL4, PLN, NPPA |
| 8 | 3.111 | 10 | 14 | COL18A1, P4HB, HSPA1A, ADORA3, AGRN, CNR2, PSAP, LRP1, LAMB2, VCAN |
| 9 | 3 | 5 | 6 | NOS1, GHRL, ADORA1, APLN, ADRB2 |
| 10 | 3 | 3 | 3 | VCL, DMD, RAC1 |
| 11 | 3 | 3 | 3 | AGTR2, APLNR, ACE2 |
| 12 | 3 | 3 | 3 | STUB1, HSPA8, DNAJA3 |
| 13 | 3 | 3 | 3 | UTS2R, GRK5, UTS2 |
| **SAP-associated networks** | | | | |
| Cluster | Score | Nodes | Edges | Gene |
| 1 | 59.383 | 82 | 2405 | CCL5, MMP1, CCL22, TNFRSF1B, CXCL13, CCL17, SPP1, PECAM1, CXCL10, MMP3, CXCR3, CCR3, CXCL12, SELE, CXCR4, PTGS2, FN1, TLR1, CASP1, EDN1, VEGFA, CCL11, EGFR, ANXA5, JUN, MPO, CCL2, CSF1, ITGAM, APOE, CRP, TLR2, CD69, IL1B, ICAM1, IL1A, CX3CL1, VCAM1, SELL, ALB, NOS3, IGF1, CXCL8, CX3CR1, MYD88, LEP, IL1R1, TNFSF11, BDNF, ANGPT2, MMP9, TLR4, IL10, ANGPT1, IL6, INS, TNF, ADIPOQ, IL4, CD40LG, ACE, MAPK1, CD40, HMOX1, TIMP1, MMP2, ELANE, TGFB1, CCL20, FOXP3, HGF, IL17A, SERPINE1, IL2, IL18, CSF3, IFNG, KDR, FGF2, CCR2, NLRP3, NOS2 |
| 2 | 11.3 | 61 | 339 | CD34, ADORA1, THBD, PNOC, LCN2, HTR1B, P2RY12, CREB1, F3, NFKB1, ELN, AGTR1, NFKBIA, HSPA4, IL7R, HIF1A, GZMB, KITLG, LGALS3, CDH5, SMAD3, CXCL16, FCGR2A, MMP7, THBS1, IFNA1, MMP10, FLT1, MMP14, FASLG, PLAUR, IDO1, CD163, VEGFC, IL33, PGF, PLAU, C5, FAS, TEK, PF4, HP, FGF1, IL3, PROM1, RETN, CAT, VWF, IL2RA, TIMP2, SELP, IL1RN, TNFRSF11B, REN, PDGFB, ADRA2A, CNR1, APLN, NOD2, CYCS, IRF1 |
| 3 | 8.129 | 32 | 126 | S100B, AGER, AHSG, MMP8, TNFRSF4, PCSK9, TNFSF4, C5AR1, ENTPD1, TNFRSF9, ADAM17, F2, APOA5, ITGB2, AGT, CP, CTSB, PLAT, TNC, SOD1, FGF23, SERPINA1, CD4, IL2RB, VTN, PROC, MFGE8, KNG1, APOB, BGLAP, SOD2, SAA1 |
| 4 | 7.31 | 30 | 106 | PPBP, FGA, SERPINC1, PTX3, OLR1, LPA, CST3, S100A9, CD63, F11, APOH, COL3A1, F2R, F5, CTSK, SHC1, CD14, ITGA2, F9, ABCA1, CETP, SORL1, PLA2G7, SAA4, FGB, APOA1, ITGB3, FABP4, RBP4, MSR1 |
| 5 | 5.667 | 7 | 17 | ADAMTS1, ADAMTS2, GDF15, ADAMTS9, ADAMTS14, ADAMTS13, ADAMTS7 |
| 6 | 4 | 4 | 6 | RARRES2, SERPINA12, ITLN1, NAMPT |
| 7 | 3.778 | 19 | 34 | HMGCR, FGF21, HSPD1, AMBP, F7, LPL, F12, S100A8, SERPINF2, HSPA1A, HLA-DRB1, PON1, APOC3, GHRL, TFPI, PPARA, GSR, MTHFR, F10 |
| 8 | 3.333 | 4 | 5 | APOA4, FETUB, HABP2, CPB2 |
| 9 | 3 | 3 | 3 | ADAMTS5, ADAMTS3, ADAMTS4 |
| 10 | 3 | 5 | 6 | NPPA, MYF5, TNNI3, MYOG, MEF2C |
| 11 | 3 | 3 | 3 | RPS27A, UBC, PIK3C2A |
| 12 | 2.667 | 4 | 4 | MB, NPPB, MYOD1, GATA4 |
| **CHF-associated networks** | | | | |
| Cluster | Score | Nodes | Edges | Gene |
| 1 | 54.137 | 103 | 2761 | ADRA2B, POMC, SELE, IL7, IL5, PECAM1, SPP1, TNFRSF1B, NPY, PDYN, APLN, ICAM1, SELP, CAT, GNB3, CCL2, MPO, HMOX1, FOXP3, CCL3, JAK2, NLRP3, PPARG, LOX, CASR, GPR17, CCL4, RLN3, CNR2, CXCR3, CXCL9, IL18, CCR4, AKT1, PENK, TNF, CXCR1, CCR5, TLR4, ADCY5, JUN, FASLG, PTGS2, GNAI1, IL17A, CASP3, EGFR, STAT3, TNFSF11, IL6R, MYD88, GAPDH, IL2, MAPK14, TNFRSF1A, MMP9, VEGFA, ADORA3, ADORA1, IL6, NGF, INS, SMAD3, MMP1, LEP, IGF1, MMP3, ALB, IL1B, VWF, THBS1, APOE, IL4, IFNG, SERPINE1, TGFB1, MMP2, TIMP1, BDNF, DRD2, HTR1A, CNR1, IL1A, HRH3, CASP1, CD274, APLNR, HIF1A, CD40, CD40LG, NOS3, STAT1, TLR9, EDN1, CTGF, FAS, HMGB1, FOS, ADIPOQ, VCAM1, CD28, ADRA2C, CSF2 |
| 2 | 18.958 | 72 | 673 | ERBB2, KDR, CD163, ADM, IL9, LGALS3, GPR29, IL22, IL11, LCN2, LTA, CALCA, NFKB1, ADORA2B, CXCL8, IL10, ENG, F3, CDH5, ELN, SMAD4, SOD2, ANGPT2, GPT, NFE2L2, NOS2, F2, NCF1, PTH, REN, TIMP2, CRP, EPO, RETN, ACE, IGFBP3, TNFRSF11B, IL1RN, VIP, SIRT1, DRD1, PTHLH, FLT1, CGA, RXFP1, CALCRL, PGF, CXCR4, CXCL12, SOD1, ADM2, RLN2, HRH2, IL33, IL2RA, HSPA4, AVPR2, CRHR2, AGTR1, MC4R, ESR1, RAMP1, RAMP2, RAMP3, ADRB1, PTEN, ADRB2, AVP, CAV1, ADAM17, CYCS, ADRB3 |
| 3 | 10.043 | 48 | 236 | ADRA1D, ADRBK1, ADRA1B, S100B, GJA1, BGLAP, CX3CR1, EDNRB, EDNRA, VDR, SERPINA1, LEPR, IL16, TGFB2, PRL, COL1A1, THBD, IL2RB, XBP1, B2M, BMP2, TGFBR1, AGT, GGT1, IRS1, LPL, DPP4, NOX1, TFRC, KNG1, PROM1, CCR1, CYBA, FOXO3, EDN3, EDN2, GRK5, AVPR1A, CYSLTR2, GNA11, HTR2B, SDHC, GHSR, PLAT, OXT, COL1A2, UTS2, GNAQ |
| 4 | 7.833 | 37 | 141 | PRKAG2, CCNA2, CTSK, ADIPOR2, ADIPOR1, PNPLA2, TNNT2, NOS1, AHSG, ACTC1, TH, SLC2A4, TF, FSTL1, TTN, LGALS1, CLU, SOD3, MYH6, PPARA, ANG, ILK, GHRL, CP, MKI67, TPM1, PARP1, MYH7, CSRP3, LDB3, FGF23, COL18A1, LMNA, SCN5A, A2M, RYR2, TNNI3 |
| 5 | 6 | 14 | 39 | HK2, NDUFB8, SDHA, FH, TPI1, SDHD, NF1, ATP5A1, G6PD, SCO1, SDHB, MT-CO3, MT-ND6, ADA |
| 6 | 5.765 | 18 | 49 | SLC8A1, AQP4, MME, NPPC, KCNJ5, KCND3, SCN1B, KCNE1, FXYD1, NPR1, ATP1B1, ATP1B3, NPR2, NPPA, ATP1A1, ATP1A2, ATP1B2, PLN |
| 7 | 5.6 | 6 | 14 | TBL2, CLIP2, RFC2, BAZ1B, GTF2IRD1, GTF2I |
| 8 | 4.286 | 8 | 15 | ACTN2, PPARGC1A, ACTN3, MYOD1, SGCD, MYH2, RBM20, MYL3 |
| 9 | 4.222 | 19 | 38 | CAV3, FBN1, MYL2, DMD, MSTN, MT-CO2, TTR, MT-CO1, NKX2-5, NPPB, COX5A, SERPINC1, ATP2A2, MYPN, DES, NDUFS2, CST3, MT-ND1, MT-ND5 |
| 10 | 4 | 4 | 6 | S100A9, CD14, S100A12, S100A8 |
| 11 | 4 | 4 | 6 | NDUFAF1, ACAD9, NDUFAF3, NDUFB11 |
| 12 | 3.455 | 12 | 19 | DLST, CPT1A, CD36, SURF1, FABP3, SDHAF2, CS, KIF1B, SLC25A3, FXN, TMEM127, MAX |
| 13 | 3.333 | 4 | 5 | BMP1, GDF2, NOG, FST |
| 14 | 3.333 | 4 | 5 | UGT1A1, CYP2C9, ABCC2, UGT2B7 |
| 15 | 3 | 3 | 3 | ERBB4, NTRK2, PSEN1 |
| 16 | 3 | 3 | 3 | MTTP, APOC2, LCAT |
| 17 | 2.833 | 13 | 17 | TRDN, RBP4, SULT2A1, RYR1, MGAM, DBH, H6PD, CACNA1C, KCNH2, APOC3, KCNQ1, KL, MGP |
| 18 | 2.667 | 7 | 8 | LIMK1, TAZ, GLA, TUBB, GLB1, VCL, FLNA |

**Table S4 Topological attributes of disease-associated modules (nodes≥4)**

| **Module** | node | edge | Avg number of neighbors | characteristic path length | network density | network heterogeneity | network centralization |
| --- | --- | --- | --- | --- | --- | --- | --- |
| ICM1 | 45 | 809 | 35.956 | 1.183 | 0.817 | 0.172 | 0.191 |
| ICM2 | 23 | 112 | 9.739 | 1.806 | 0.443 | 0.462 | 0.361 |
| ICM3 | 13 | 55 | 8.462 | 1.308 | 0.705 | 0.205 | 0.25 |
| ICM4 | 10 | 19 | 3.8 | 1.911 | 0.422 | 0.404 | 0.306 |
| ICM5 | 4 | 6 | 3 | 1 | 1 | 0 | 0 |
| ICM6 | 8 | 12 | 3 | 1.821 | 0.429 | 0.236 | 0.19 |
| ICM7 | 4 | 5 | 2.5 | 1.167 | 0.833 | 0.2 | 0.333 |
| ICM8 | 10 | 14 | 2.8 | 2.089 | 0.311 | 0.35 | 0.306 |
| ICM9 | 5 | 6 | 2.4 | 1.4 | 0.6 | 0.204 | 0.25 |
| SAP1 | 82 | 2405 | 58.659 | 1.276 | 0.724 | 0.235 | 0.283 |
| SAP2 | 61 | 339 | 11.115 | 2.084 | 0.185 | 0.405 | 0.256 |
| SAP3 | 32 | 126 | 7.875 | 2.313 | 0.254 | 0.618 | 0.314 |
| SAP4 | 30 | 106 | 7.067 | 2.177 | 0.244 | 0.542 | 0.293 |
| SAP5 | 7 | 17 | 4.857 | 1.19 | 0.81 | 0.256 | 0.267 |
| SAP6 | 4 | 6 | 3 | 1 | 1 | 0 | 0 |
| SAP7 | 19 | 34 | 3.579 | 2.608 | 0.199 | 0.343 | 0.15 |
| SAP8 | 4 | 5 | 2.5 | 1.167 | 0.833 | 0.2 | 0.333 |
| SAP10 | 5 | 6 | 2.4 | 1.4 | 0.6 | 0.333 | 0.667 |
| SAP12 | 4 | 4 | 2 | 1.333 | 0.667 | 0 | 0 |
| CHF1 | 103 | 2761 | 53.612 | 1.477 | 0.526 | 0.314 | 0.374 |
| CHF2 | 72 | 673 | 18.694 | 1.878 | 0.263 | 0.456 | 0.454 |
| CHF3 | 48 | 236 | 9.833 | 2.312 | 0.209 | 0.713 | 0.315 |
| CHF4 | 37 | 141 | 7.622 | 2.605 | 0.212 | 0.562 | 0.187 |
| CHF5 | 14 | 39 | 5.571 | 1.67 | 0.429 | 0.443 | 0.487 |
| CHF6 | 18 | 49 | 5.444 | 2.026 | 0.32 | 0.353 | 0.235 |
| CHF7 | 6 | 14 | 4.667 | 1.067 | 0.933 | 0.101 | 0.1 |
| CHF8 | 8 | 15 | 3.75 | 1.607 | 0.536 | 0.291 | 0.238 |
| CHF9 | 19 | 38 | 4 | 3.48 | 0.222 | 0.344 | 0.124 |
| CHF10 | 4 | 6 | 3 | 1 | 1 | 0 | 0 |
| CHF11 | 4 | 6 | 3 | 1 | 1 | 0 | 0 |
| CHF12 | 12 | 19 | 3.167 | 2.333 | 0.288 | 0.404 | 0.418 |
| CHF13 | 4 | 5 | 2.5 | 1.167 | 0.833 | 0.2 | 0.333 |
| CHF14 | 4 | 5 | 2.5 | 1.167 | 0.833 | 0.2 | 0.333 |
| CHF17 | 13 | 17 | 2.615 | 3.128 | 0.218 | 0.282 | 0.136 |
| CHF18 | 7 | 8 | 2.286 | 2.19 | 0.381 | 0.198 | 0.167 |

**Table S5 Reconfiguration module matching among ICM-, SAP- and CHF--associated networks**

| ICM vs SAP | | |
| --- | --- | --- |
| ICM | SAP | Overlapping gene |
| ICM1 | SAP1 | ANGPT2, CSF3, IL18, VEGFA, EDN1, FN1, MMP3, MMP1, FGF2, KDR, SERPINE1, HGF, TGFB1, MMP2, TIMP1, ACE, TNF, INS, IL6, IL10, CXCL8, IGF1, NOS3, VCAM1, ICAM1, CRP, CCL2 |
| ICM1 | SAP2 | CDH5, PGF, TEK, HIF1A, FLT1, MMP14, REN, VWF, THBS1, VEGFC, PDGFB, SELP |
| ICM1 | SAP3 | AGT |
| ICM2 | SAP3 | TNC, FGF23 |
| ICM2 | SAP4 | APOA1 |
| ICM3 | SAP4 | COL3A1 |
| ICM4 | SAP12 | NPPB |
| ICM5 | SAP5 | ADAMTS9 |
| ICM6 | SAP2 | AGTR1, KITLG |
| ICM6 | SAP3 | APOB |
| ICM7 | SAP10 | NPPA |
| ICM8 | SAP7 | HSPA1A |
| ICM9 | SAP2 | ADORA1, APLN |
| ICM9 | SAP7 | GHRL |
| SAP vs CHF | | |
| SAP | CHF | Overlapping gene |
| SAP1 | CHF1 | IL17A, MMP2, BDNF, TLR4, IFNG, ALB, EDN1, IL4, SERPINE1, IGF1, VCAM1, HMOX1, INS, TNF, VEGFA, PECAM1, TGFB1, MYD88, IL1A, MMP3, ICAM1, JUN, CD40, TNFRSF1B, MPO, TIMP1, NLRP3, LEP, CASP1, CCL2, SPP1, CD40LG, MMP1, NOS3, IL6, PTGS2, FOXP3, TNFSF11, EGFR, IL1B, SELE, IL18, CXCR3, APOE, MMP9, IL2, ADIPOQ |
| SAP1 | CHF2 | ACE, CXCR4, KDR, CXCL12, NOS2, IL10, CRP, CXCL8, ANGPT2 |
| SAP1 | CHF3 | CX3CR1 |
| SAP2 | CHF1 | FASLG, THBS1, CAT, SELP, APLN, HIF1A, SMAD3, VWF, CNR1, ADORA1, FAS |
| SAP2 | CHF2 | LCN2, LGALS3, AGTR1, RETN, F3, HSPA4, CDH5, PGF, CD163, TNFRSF11B, CYCS, FLT1, IL33, NFKB1, TIMP2, REN, IL1RN, IL2RA, ELN |
| SAP2 | CHF3 | THBD, PROM1 |
| SAP3 | CHF2 | SOD2, F2, SOD1, ADAM17 |
| SAP3 | CHF3 | IL2RB, SERPINA1, BGLAP, AGT, KNG1, PLAT, S100B |
| SAP3 | CHF4 | CP, AHSG, FGF23 |
| SAP4 | CHF4 | CTSK |
| SAP4 | CHF9 | CST3, SERPINC1 |
| SAP4 | CHF10 | CD14, S100A9 |
| SAP4 | CHF17 | RBP4 |
| SAP7 | CHF3 | LPL |
| SAP7 | CHF4 | GHRL, PPARA |
| SAP7 | CHF10 | S100A8 |
| SAP7 | CHF17 | APOC3 |
| SAP10 | CHF4 | TNNI3 |
| SAP10 | CHF6 | NPPA |
| SAP12 | CHF8 | MYOD1 |
| SAP12 | CHF9 | NPPB |
| ICM vs CHF | | |
| ICM | CHF | Overlapping gene |
| ICM1 | CHF1 | IL18, THBS1, HIF1A, NOS3, EDN1, IL6, TNF, VCAM1, IGF1, MMP3, MMP1, ICAM1, SELP, VWF, CCL2, SERPINE1, TGFB1, MMP2, TIMP1, PPARG, VEGFA, INS, CASP3 |
| ICM1 | CHF2 | CDH5, PGF, FLT1, ANGPT2, IL10, REN, C RP, ACE, CXCL8, ERBB2, KDR |
| ICM1 | CHF3 | AGT |
| ICM2 | CHF3 | GJA1，COL1A1，COL1A2 |
| ICM2 | CHF4 | LGALS1, TF, FGF23 |
| ICM4 | CHF4 | SCN5A, TNNT2, LMNA |
| ICM4 | CHF9 | ATP2A2, CAV3, NPPB |
| ICM6 | CHF1 | STAT1 |
| ICM6 | CHF2 | IGFBP3, AGTR1 |
| ICM6 | CHF4 | ANG |
| ICM7 | CHF6 | PLN, NPPA |
| ICM7 | CHF9 | MYL2 |
| ICM8 | CHF1 | ADORA3, CNR2 |
| ICM8 | CHF4 | COL18A1 |
| ICM9 | CHF1 | ADORA1, APLN |
| ICM9 | CHF2 | ADRB2 |
| ICM9 | CHF4 | GHRL, NOS1 |

**Table S6** **Weight results calculated by entropy method**

| Items | Information entropy | Information utility value | weight coefficient |
| --- | --- | --- | --- |
| node | 0.8775 | 0.1225 | 11.72% |
| edge | 0.7844 | 0.2156 | 20.63% |
| Avg number of neighbors | 0.839 | 0.161 | 15.41% |
| characteristic path length | 0.8869 | 0.1131 | 10.83% |
| network density | 0.8893 | 0.1107 | 10.59% |
| network heterogeneity | 0.8831 | 0.1169 | 11.19% |
| network centralization | 0.7948 | 0.2052 | 19.63% |

**Table S7 Normalization of disease-related module parameters (nodes≥4)**

| Module | Node | Edge | Avg number of neighbors | characteristic path length |
| --- | --- | --- | --- | --- |
| ICM1 | 1 | 1 | 1 | 0.832 |
| ICM2 | 0.463 | 0.133 | 0.219 | 0.260 |
| ICM3 | 0.220 | 0.062 | 0.181 | 0.717 |
| ICM4 | 0.146 | 0.017 | 0.042 | 0.163 |
| ICM5 | 0 | 0.001 | 0.018 | 1 |
| ICM6 | 0.098 | 0.009 | 0.018 | 0.246 |
| ICM7 | 0 | 0 | 0.003 | 0.847 |
| ICM8 | 0.146 | 0.011 | 0.012 | 0 |
| ICM9 | 0.024 | 0.001 | 0 | 0.633 |
| SAP1 | 1.000 | 1.000 | 1.000 | 0.828 |
| SAP2 | 0.731 | 0.140 | 0.161 | 0.326 |
| SAP3 | 0.359 | 0.051 | 0.104 | 0.183 |
| SAP4 | 0.333 | 0.042 | 0.089 | 0.268 |
| SAP5 | 0.038 | 0.005 | 0.050 | 0.882 |
| SAP6 | 0.000 | 0.001 | 0.018 | 1.000 |
| SAP7 | 0.192 | 0.012 | 0.028 | 0.000 |
| SAP8 | 0.000 | 0.000 | 0.009 | 0.896 |
| SAP10 | 0.013 | 0.001 | 0.007 | 0.751 |
| SAP12 | 0.000 | 0.000 | 0.000 | 0.793 |
| CHF1 | 1.000 | 1.000 | 1.000 | 0.808 |
| CHF2 | 0.687 | 0.242 | 0.320 | 0.646 |
| CHF3 | 0.444 | 0.084 | 0.147 | 0.471 |
| CHF4 | 0.333 | 0.049 | 0.104 | 0.353 |
| CHF5 | 0.101 | 0.012 | 0.064 | 0.730 |
| CHF6 | 0.141 | 0.016 | 0.062 | 0.586 |
| CHF7 | 0.020 | 0.003 | 0.046 | 0.973 |
| CHF8 | 0.040 | 0.004 | 0.029 | 0.755 |
| CHF9 | 0.152 | 0.012 | 0.033 | 0.000 |
| CHF10 | 0.000 | 0.000 | 0.014 | 1.000 |
| CHF11 | 0.000 | 0.000 | 0.014 | 1.000 |
| CHF12 | 0.081 | 0.005 | 0.017 | 0.463 |
| CHF13 | 0.000 | 0.000 | 0.004 | 0.933 |
| CHF14 | 0.000 | 0.000 | 0.004 | 0.933 |
| CHF17 | 0.091 | 0.004 | 0.006 | 0.142 |
| CHF18 | 0.030 | 0.001 | 0.000 | 0.520 |

**Table S8 K-value of reconstruction module pairs among ICM, SAP and CHF disease (nodes≥4)**

| MRP | k-value | Statistical distribution | d1 | d2 | d3 | d4 | d5 | d6 | d7 |
| --- | --- | --- | --- | --- | --- | --- | --- | --- | --- |
| MSAP2-MCHF1 | 0.406 | 100.0% | 0.038 | 0.392 | 0.373 | 0.123 | 0.062 | 0.004 | 0.007 |
| MSAP1-MCHF2 | 0.404 | 98.1% | 0.067 | 0.393 | 0.317 | 0.023 | 0.146 | 0.033 | 0.020 |
| MSAP7-MICM8 | 0.403 | 97.0% | 0.054 | 0.000 | 0.006 | 0.000 | 0.319 | 0.001 | 0.619 |
| MSAP10-MCHF6 | 0.401 | 94.4% | 0.053 | 0.001 | 0.009 | 0.087 | 0.251 | 0.001 | 0.597 |
| MICM1-MCHF2 | 0.400 | 92.3% | 0.060 | 0.353 | 0.285 | 0.021 | 0.189 | 0.050 | 0.043 |
| MICM9-MCHF1 | 0.399 | 91.0% | 0.316 | 0.331 | 0.332 | 0.010 | 0.002 | 0.004 | 0.005 |
| MSAP10-MICM7 | 0.398 | 90.5% | 0.001 | 0.000 | 0.000 | 0.047 | 0.282 | 0.092 | 0.579 |
| MSAP4-MCHF4 | 0.397 | 89.1% | 0.000 | 0.002 | 0.011 | 0.358 | 0.051 | 0.020 | 0.559 |
| MICM6-MCHF1 | 0.395 | 86.4% | 0.260 | 0.314 | 0.309 | 0.101 | 0.003 | 0.002 | 0.011 |
| MSAP1-MCHF3 | 0.394 | 85.3% | 0.124 | 0.336 | 0.291 | 0.051 | 0.106 | 0.091 | 0.000 |
| MSAP2-MICM1 | 0.394 | 84.7% | 0.032 | 0.332 | 0.316 | 0.115 | 0.179 | 0.024 | 0.002 |
| MICM1-MCHF3 | 0.391 | 80.6% | 0.115 | 0.313 | 0.271 | 0.049 | 0.138 | 0.109 | 0.006 |
| MICM8-MCHF1 | 0.389 | 78.5% | 0.215 | 0.289 | 0.288 | 0.193 | 0.014 | 0.000 | 0.001 |
| MSAP3-MICM1 | 0.388 | 76.7% | 0.134 | 0.294 | 0.262 | 0.137 | 0.103 | 0.065 | 0.005 |
| MSAP1-MICM1 | 0.378 | 64.2% | 0.000 | 0.000 | 0.000 | 0.001 | 0.410 | 0.188 | 0.401 |
| MSAP5-MICM5 | 0.377 | 62.8% | 0.008 | 0.000 | 0.006 | 0.074 | 0.191 | 0.346 | 0.376 |
| MSAP12-MCHF8 | 0.375 | 60.1% | 0.010 | 0.000 | 0.005 | 0.009 | 0.106 | 0.522 | 0.349 |
| MSAP10-MCHF4 | 0.373 | 56.4% | 0.145 | 0.003 | 0.013 | 0.225 | 0.213 | 0.074 | 0.326 |
| MSAP2-MCHF2 | 0.372 | 55.1% | 0.010 | 0.056 | 0.134 | 0.545 | 0.032 | 0.014 | 0.208 |
| MICM2-MCHF4 | 0.370 | 53.2% | 0.121 | 0.050 | 0.094 | 0.062 | 0.383 | 0.072 | 0.217 |
| MSAP3-MCHF4 | 0.369 | 52.2% | 0.013 | 0.000 | 0.000 | 0.569 | 0.035 | 0.062 | 0.320 |
| MICM4-MCHF9 | 0.368 | 50.6% | 0.000 | 0.000 | 0.001 | 0.258 | 0.386 | 0.035 | 0.320 |
| MICM6-MCHF2 | 0.363 | 43.6% | 0.435 | 0.068 | 0.114 | 0.200 | 0.035 | 0.061 | 0.087 |
| MSAP4-MICM2 | 0.361 | 41.3% | 0.183 | 0.089 | 0.181 | 0.001 | 0.428 | 0.069 | 0.050 |
| MICM9-MCHF2 | 0.361 | 40.6% | 0.536 | 0.071 | 0.125 | 0.000 | 0.139 | 0.078 | 0.051 |
| MICM1-MCHF1 | 0.359 | 37.9% | 0.000 | 0.000 | 0.000 | 0.004 | 0.610 | 0.145 | 0.241 |
| MSAP4-MCHF17 | 0.357 | 35.8% | 0.334 | 0.008 | 0.039 | 0.090 | 0.004 | 0.384 | 0.140 |
| MSAP3-MCHF2 | 0.356 | 34.9% | 0.239 | 0.081 | 0.104 | 0.475 | 0.000 | 0.058 | 0.043 |
| MSAP4-MCHF9 | 0.355 | 33.2% | 0.187 | 0.005 | 0.018 | 0.405 | 0.003 | 0.221 | 0.161 |
| MSAP3-MICM2 | 0.352 | 29.5% | 0.110 | 0.068 | 0.134 | 0.059 | 0.361 | 0.246 | 0.022 |
| MSAP1-MCHF1 | 0.347 | 22.7% | 0.000 | 0.000 | 0.000 | 0.008 | 0.724 | 0.115 | 0.153 |
| MSAP12-MICM4 | 0.347 | 22.4% | 0.029 | 0.000 | 0.002 | 0.538 | 0.081 | 0.222 | 0.127 |
| MSAP2-MICM6 | 0.347 | 22.4% | 0.746 | 0.032 | 0.038 | 0.012 | 0.111 | 0.053 | 0.008 |
| MICM4-MCHF4 | 0.346 | 21.1% | 0.220 | 0.006 | 0.024 | 0.226 | 0.277 | 0.157 | 0.089 |
| MSAP3-MICM6 | 0.345 | 19.9% | 0.250 | 0.006 | 0.027 | 0.014 | 0.112 | 0.534 | 0.056 |
| MSAP7-MCHF3 | 0.343 | 17.9% | 0.136 | 0.011 | 0.030 | 0.473 | 0.000 | 0.292 | 0.058 |
| MICM8-MCHF4 | 0.343 | 16.9% | 0.147 | 0.006 | 0.036 | 0.522 | 0.041 | 0.189 | 0.059 |
| MSAP2-MICM9 | 0.342 | 15.4% | 0.586 | 0.022 | 0.030 | 0.111 | 0.202 | 0.047 | 0.000 |
| MSAP2-MCHF3 | 0.341 | 15.0% | 0.399 | 0.015 | 0.001 | 0.103 | 0.003 | 0.462 | 0.017 |
| MICM6-MCHF4 | 0.337 | 9.9% | 0.242 | 0.007 | 0.032 | 0.050 | 0.205 | 0.463 | 0.000 |
| MSAP4-MCHF10 | 0.337 | 9.5% | 0.069 | 0.001 | 0.004 | 0.334 | 0.356 | 0.183 | 0.053 |
| MICM2-MCHF3 | 0.336 | 7.6% | 0.002 | 0.014 | 0.030 | 0.259 | 0.318 | 0.366 | 0.012 |
| MICM9-MCHF4 | 0.336 | 7.4% | 0.203 | 0.005 | 0.023 | 0.167 | 0.321 | 0.273 | 0.008 |
| MSAP7-MCHF4 | 0.335 | 7.1% | 0.099 | 0.007 | 0.029 | 0.619 | 0.001 | 0.239 | 0.007 |
| MSAP7-MCHF17 | 0.335 | 6.9% | 0.292 | 0.002 | 0.013 | 0.572 | 0.010 | 0.106 | 0.006 |
| MICM7-MCHF9 | 0.333 | 4.2% | 0.019 | 0.000 | 0.001 | 0.608 | 0.317 | 0.018 | 0.037 |
| MSAP3-MCHF3 | 0.333 | 3.9% | 0.070 | 0.010 | 0.018 | 0.795 | 0.019 | 0.087 | 0.000 |
| MICM7-MCHF6 | 0.332 | 2.3% | 0.052 | 0.001 | 0.009 | 0.175 | 0.679 | 0.060 | 0.025 |
| MSAP12-MCHF9 | 0.331 | 1.8% | 0.023 | 0.000 | 0.001 | 0.639 | 0.201 | 0.120 | 0.016 |
| MSAP7-MICM9 | 0.331 | 1.4% | 0.046 | 0.000 | 0.001 | 0.646 | 0.260 | 0.031 | 0.016 |
| MSAP4-MICM3 | 0.331 | 1.0% | 0.023 | 0.001 | 0.015 | 0.366 | 0.385 | 0.206 | 0.003 |
| MSAP7-MCHF10 | 0.330 | 0.0% | 0.020 | 0.000 | 0.000 | 0.550 | 0.353 | 0.065 | 0.012 |

d1=node; d2=edge; d3=Avg number of neighbors; d4=characteristic path length; d5=Network density; d6=network heterogeneity; d7=Network centralization

**Table S9 Overlapping pathways and biological processes at each stage based on PDPMs**

| Stage | Overlapping KEGG | Overlapping GO biological processes |
| --- | --- | --- |
| SAP-CHF | AGE-RAGE signaling pathway in diabetic complications | response to molecule of bacterial origin |
|  | Chagas disease (American trypanosomiasis) | cytokine-mediated signaling pathway |
|  | foxo signaling pathway | positive regulation of response to external stimulus |
|  | Adipocytokine signaling pathway | response to wounding |
|  | Apelin signaling pathway | regulation of secretion by cell |
|  | Cytokine-cytokine receptor interaction | apoptotic signaling pathway |
|  | Hepatitis B | multicellular organismal homeostasis |
|  | Complement and coagulation cascades | regulation of body fluid levels |
|  | Fluid shear stress and atherosclerosis | response to oxygen levels |
|  |  | positive regulation of cytokine production |
| ICM-CHF | AGE-RAGE signaling pathway in diabetic complications | positive regulation of pri-miRNA transcription by RNA polymerase II |
|  | p53 signaling pathway | negative regulation of cell population proliferation |
|  | Longevity regulating pathway | regulation of smooth muscle cell proliferation |
|  | Neuroactive ligand-receptor interaction |  |
| SAP-ICM | PI3K-Akt signaling pathway | positive regulation of cell migration |
|  | Hypertrophic cardiomyopathy (HCM) | negative regulation of response to external stimulus |
|  | Staphylococcus aureus infection | response to hypoxia |
|  |  | tissue morphogenesis |
|  |  | extracellular matrix organization |

**Table S10 Literature validation of top 3 pathways in three diseases**

|  | KEGG pathway | Function | Literature |
| --- | --- | --- | --- |
| SAP | cytokine-cytokine receptor interaction | - | - |
|  | complement and coagulation cascades | Venous thrombosis and atherosclerosis | Wang P,etal,2020 |
|  | malaria | - | - |
| ICM | Focal adhesion | Inhibition of ventricular remodeling | Wang L,etal,2013;Liu XL,2013 |
|  | AGE-RAGE signaling pathway in diabetic complications | Activate fibroblasts | Cao J,etal,2021 |
|  | pathways in cancer | - | - |
| CHF | cytokine-cytokine receptor interaction | Immune-related and metabolic | Li J,etal,2020 |
|  | Neuroactive ligand-receptor interaction | Cardiac function | Wang Y,etal,2014 |
|  | Pathways in cancer | Angiogenesis etal | de Boer RA.etal.2020 |

**Table S11 Overlapping KEGG pathways in SAP-ICM-CHF in PDPMS**

|  | Pathway | SAP | ICM | CHF | Literature |
| --- | --- | --- | --- | --- | --- |
| Malaria | Malaria | X | √ | X | Guan YZ etal， 2020 |
| Cancer | Pathways in cancer | x | X | √ | de Boer RA etal，2020；Qian C etal，2018 |
|  | Bladder cancer | X | X | X |  |
|  | Transcriptional misregulation in cancer | x | x | x |  |
| Oxidative stress | HIF-1 signaling pathway | √ | √ | √ | Tao YG etal，2020；Bourdier G etal，2020；Liu Q，2013 |
| Inflammation | Leukocyte transendothelial migration | X | X | X |  |

Reference:

Bourdier G, Détrait M, Bouyon S, et al. Intermittent Hypoxia Triggers Early Cardiac Remodeling and Contractile Dysfunction in the Time-Course of Ischemic Cardiomyopathy in Rats. J Am Heart Assoc. 2020;9(16):e016369.

Cao J, Liu Z, Liu J, Li C, Zhang G, Shi R. Bioinformatics Analysis and Identification of Genes and Pathways in Ischemic Cardiomyopathy. Int J Gen Med. 2021 Sep 21;14:5927-5937.

de Boer RA, Hulot JS, Tocchetti CG,etal. Common mechanistic pathways in cancer and heart failure. A scientific roadmap on behalf of the Translational Research Committee of the Heart Failure Association (HFA) of the European Society of Cardiology (ESC). Eur J Heart Fail. 2020 Dec;22(12):2272-2289.

Guan YZ, Yin RX, Deng GX, Zheng PF, Liu CX, Wei BL. Potential Molecular Mechanism of the NPPB Gene in Postischemic Heart Failure with and without T2DM. Biomed Res Int. 2020;2020:2159460. Published 2020 Aug 3.

Liu XL, Li G, Wang ZH, Zhao WJ, Wang LP. Increased expression of Dock180 protein in the noninfarcted myocardium in rats. J Chin Med Assoc. 2013 Mar;76(3):164-8.

Liu Q, Liang Y, Zou P, Ni WX, Li YG, Chen SM. Hypoxia-inducible factor-1α polymorphisms link to coronary artery collateral development and clinical presentation of coronary artery disease. Biomed Pap Med Fac Univ Palacky Olomouc Czech Repub. 2013;157(4):340-345.

Li J, Su H, Zhu Y, Cao Y, Ma X. ETS2 and microRNA-155 regulate the pathogenesis of heart failure through targeting and regulating GPR18 expression. Exp Ther Med. 2020 Jun;19(6):3469-3478.

Qian C, Chang D, Li H, Wang Y. Identification of potentially critical genes in the development of heart failure after ST-segment elevation myocardial infarction (STEMI) [published online ahead of print, 2018 Nov 28]. J Cell Biochem. 2018;10.1002/jcb.28051.

Tao YG, Huang XF, Wang JY, Kang MR, Wang LJ, Xian SX. Exploring Molecular Mechanism of Huangqi in Treating Heart Failure Using Network Pharmacology. Evid Based Complement Alternat Med. 2020;2020:6473745. Published 2020 Apr 23.

Wang P, Huang H, Chen B, Su Y, Shi P, Yao H. Systems Pharmacology Dissection of Mechanisms of Dengzhan Xixin Injection against Cardiovascular Diseases. Chem Pharm Bull (Tokyo). 2020;68(9):837-847.

Wang L, Li G, Wang Z, Liu X, Zhao W. Elevated expression of C3G protein in the peri-infarct myocardium of rats. Med Sci Monit Basic Res. 2013 Jan 1;19:1-5.

Wang Y, Li C, Liu Z, Shi T, Wang Q, Li D, Wu Y, Han J, Guo S, Tang B, Wang W. DanQi Pill protects against heart failure through the arachidonic acid metabolism pathway by attenuating different cyclooxygenases and leukotrienes B4. BMC Complement Altern Med. 2014 Feb 20;14:67.
